# Ultrasound scanning for percutaneous dilatational tracheostomy: a systematic review

**DOI:** 10.1186/cc12101

**Published:** 2013-03-19

**Authors:** R Pugh, A Slater

**Affiliations:** 1 Glan Clwyd Hospital, Bodelwyddan, UK

## Introduction

Percutaneous dilatational tracheostomy (PDT) remains a frequently performed procedure in the ICU. However, there is great variability in the course of blood vessels in the pre-tracheal area. A 5% risk of clinically relevant bleeding was recently reported for patients undergoing PDT [1]. We conducted a systematic review of reports evaluating clinical outcomes following use of ultrasound scanning (US) for PDT.

## Methods

Two investigators performed a search of the literature using the following databases: CENTRAL, Embase, MEDLINE and SCOPUS. The following eligibility criteria were used: population including adults >16 years managed in the ICU; use of ultrasound to guide decisionmaking pre-PDT or guide PDT performance; report of clinically relevant outcome measures. Nonrandomised controlled trials were classified according to Cochrane Non-Randomised Study Methods Group criteria [2] and evaluated for risk of bias.

## Results

An initial search identified 2,043 reports, of which 10 studies met eligibility criteria: eight case series, one randomised controlled trial (RCT) and one prospective cohort study, incorporating 488 patients. Two studies specifically reported data on patients with obesity (*n = *29 patients) and one study reported data for a group of patients with spinal cord fixation (*n = *6). US was used to guide decision to perform PDT or surgical tracheostomy in five studies, with decision to perform surgical tracheostomy ranging from 0 to 27% of cases. US was used to guide insertion point in seven studies, and used real-time in four studies. Times to perform US-guided PDT were reported in four studies (ranging from 8 to 12 minutes). No studies compared time taken with or without US. Data on complications of procedure were reported in nine studies. Minor bleeding was reported for eight cases (1.6% overall). Prolonged bleeding was reported in two cases (0.4%). There were no episodes of catastrophic bleeding among 488 cases. High risk of bias was identified in five studies in terms of patient selection. An intervention protocol was not defined in three reports. No attempt was made at blinding any aspect of the 10 studies.

## Conclusion

Use of US guidance could theoretically help minimise risk of haemorrhagic complications during PDT and perhaps reduce time taken to perform PDT. However, there is currently inadequate evidence from controlled cohort studies or RCTs to suggest that routine use for PDT in selected or unselected groups improves clinically relevant outcome measure.
